# Expert Consensus on the Application of Stem Cells in Psoriasis Research and Clinical Trials

**DOI:** 10.14336/AD.2024.0012

**Published:** 2025-06-04

**Authors:** Ye-Hong Kuang, Wu Zhu, Ge Lin, La-Mei Cheng, Qun Qin, Zhi-Jun Huang, Yu-Ling Shi, Chun-Lei Zhang, Jin-Hua Xu, Ke-Xiang Yan, Cheng-Zhi Lv, Wei Li, Qin Han, Ilia Stambler, Lee Wei Lim, Sasanka Chakrabarti, Brun Ulfhake, Kyung-Jin Min, Georgina Ellison-Hughes, William C Cho, Kunlin Jin, Danni Yao, Chuanjian Lu, Robert Chunhua Zhao, Xiang Chen

**Affiliations:** ^1^ Department of Dermatology, Xiangya Hospital, Central South University, Changsha, China.; ^2^ Institute of Reproductive and Stem Cell Engineering, School of Basic Medical Science, Central South University, Changsha, China.; ^3^ National Engineering Research Center of Human Stem Cell, Changsha, China.; ^4^ The Office of Drug Clinical Trials Institution, Xiangya Hospital, Central South University, Changsha, China.; ^5^ Center for Clinical Pharmacology, The Third Xiangya Hospital of Central South University, Changsha, China.; ^6^ Department of Dermatology, Shanghai Skin Disease Hospital, Institute of Psoriasis, School of Medicine, Tongji University, Shanghai, China.; ^7^ Department of Dermatology, Peking University Third Hospital, Beijing, China.; ^8^ Department of Dermatology, Huashan Hospital, Fudan University, Shanghai, China.; ^9^ Department of Dermatology, Dalian Dermatosis Hospital, Dalian, China.; ^10^ Department of Dermatology, Rare Diseases Center, West China Hospital, Sichuan University, Chengdu, China.; ^11^ International Society on Aging and Disease, Fort Worth, TX, USA.; ^12^ Department of Science, Technology and Society, Bar Ilan University, Ramat Gan, Israel.; ^13^ School of Biomedical Sciences, Li Ka Shing Faculty of Medicine, University of Hong Kong, Hong Kong, China.; ^14^ Maharishi Markandeshwar Deemed University, Mullana-Ambala, India.; ^15^ Karolinska University Hospital, Stockholm, Sweden.; ^16^ Department of Biological Sciences, Inha University, Incheon, Republic of Korea.; ^17^ School of Basic and Medical Biosciences, Faculty of Life Sciences & Medicine, King’s College London, London, UK.; ^18^ Department of Clinical Oncology, Queen Elizabeth Hospital, Hong Kong SAR, China.; ^19^ University of North Texas Health Science Center, Bryan, TX, USA.; ^20^ Institute of Basic Medical Sciences, Chinese Academy of Medical Sciences, School of Basic Medicine, Peking Union Medical College, Beijing, China.; ^21^ Center for Excellence in Tissue Engineering, Chinese Academy of Medical Sciences, Beijing, China.; ^22^ Beijing Key Laboratory of New Drug Development and Clinical Trial of Stem Cell Therapy (BZ0381), Beijing, China.; ^23^ Department of Dermatology, Guangdong Provincial Hospital of Chinese Medicine, Guangzhou, China.; ^24^ State Key Laboratory of Dampness Syndrome of Chinese Medicine, The Second Affiliated Hospital of Guangzhou University of Chinese Medicine (Guangdong Provincial Hospital of Chinese Medicine), Guangzhou, China.; ^25^ Guangdong Provincial Key Laboratory of Clinical Research on Traditional Chinese Medicine Syndrome, The Second Affiliated Hospital of Guangzhou University of Chinese Medicine, Guangzhou, China.; ^26^ Guangdong-Hong Kong-Macau Joint Lab on Chinese Medicine and Immune Disease Research, Guangzhou University of Chinese Medicine, Guangzhou, China.; ^27^ China Dermatologist Association, China.; ^28^ Chinese Society of Dermatology, China.

**Keywords:** psoriasis, stem cell, treatment, consensus

## Abstract

Psoriasis is an immune-mediated, chronic, relapsing, inflammatory, systemic disease induced by individual-environmental interactions, and is often lifelong because of the difficulty of treatment. In recent years, a variety of targeted therapies, including biologics, have improved the lesions and quality of life of most psoriasis patients, but they still do not address the problem of relapse and may be associated with decreased efficacy or adverse events such as infections over time. Therefore, there is an urgent need for breakthroughs in psoriasis treatment and in relapse-delaying and non-pharmacologic strategies, and stem cell therapy for psoriasis has emerged. In recent years, research on stem cell therapy for psoriasis has received a lot of attention, however, there is no reference standard as well as consensus in this field of research. Therefore, according to the latest consensus and guidelines, combined with relevant literature reports, clinical practice experience and the results of discussions with experts, this consensus specifies the types of stem cells commonly used in the treatment of psoriasis, the methods, dosages, and routes of stem cell therapy for psoriasis, as well as the clinical evaluations (efficacy and safety) of stem cell therapy for psoriasis. In addition, this consensus also provides normative standards for the processes of collection, preparation, preservation and quality control of stem cells and their related products, as well as recommendations for the management of stem cells during infusion for the treatment of psoriasis. This consensus provides the latest specific reference standards and practice guidelines for the field of stem cell therapy for psoriasis.

## 1. General rules

Stem cells, obtained from embryonic tissues or adult tissues, have both self-renewal ability and differentiation potential. They can be autologous or allogeneic and isolated and passaged in vitro, fresh or cryopreserved.

### 1.1 Requirements for carrying out preclinical and clinical research with stem cells

#### 1.1.1 Requirements for preclinical stem cell research.

Researchers should rigorously develop stem cell research protocols and ensure that records and reports are comprehensive, accurate, and maintain a clear audit trail to facilitate verification and accountability. This ensures that trials meet scientific and medical requirements and provide a sufficient scientific basis for stem cell clinical trials. The requirements for the cells used are the same as those for clinical cells (cells are of quality and purity suitable for use in clinical applications). The production and processing of cell products must be carried out in an environment that adheres to good manufacturing practices (GMPs). In addition, genome stability, tumorigenesis/ tumorigenicity (the ability of a substance or process to cause the formation of tumors), abnormal differentiation and biological analyses should be performed to identify biological functions and effects. The selection of animal models must follow the three principles: reduction, refinement, and replacement. The design principles of efficacy studies include simulating clinical trial conditions, sufficient statistical power, sufficient controls, randomization, and blinding. Meanwhile, establishing a dose-response relationship is also one of the design principles for efficacy studies. Safety studies should include the monitoring of cell biodistribution, abnormal and ectopic distribution and differentiation, and other short- and long-term possible toxic side effects.

#### 1.1.2 Requirements for clinical research on stem cells.

Stem cell clinical research must have a sufficient scientific basis, and the expected benefits and risks to the health of the subjects and the public should be weighed. The benefits should outweigh the possible harms. The well-being and rights of individuals participating in clinical research on stem cells must be safeguarded, encompassing studies that involve stem cell-derived treatments and innovative reproductive technologies.

According to the ISSCR’s Guidelines for Stem Cell Research and Clinical Translation [1], all studies involving the clinical application of stem cell-based interventions must undergo prospective review and be approved by an independent Human Subjects Research Review Committee. The review process for stem cell clinical studies should ensure that protocols are supervised by independent experts. These experts have the ability to (i) evaluate preclinical and clinical studies related to stem cells; (ii) complete the design of stem cell clinical studies, including the adequacy of planned analytical endpoints, statistical considerations, and disease-specific issues related to the protection of human subjects. After the end of the study, subjects should be monitored by long-term follow-up to evaluate the long-term safety and efficacy of stem cell treatments.

### 1.2 Requirements for institutions that manufacture stem cells and related products

1.2.1

Institutions that prepare stem cells and related products (hereinafter referred to as institutions) should have a GMP production workshop that meets the following standards: overall level, 10,000; local level, 100; layout that meets drug GMP requirements; and a standard operation procedure (SOP) for each instrument.

1.2.2

Institutions should establish a complete quality management system for stem cell preparation that meets the GMP requirements and establish an independent quality management department to perform quality assurance and quality control responsibilities. Institutions should perform risk assessments based on the characteristics and preparation processes for each stem cell medical product.

1.2.3

Institutions should establish a reasonable quality management strategy and designate a specific person for stem cell preparation management, a person for quality management and a person for quality. Additionally, personnel and equipment management files should be established; relevant personnel should be provided with professional knowledge, safety protection, emergency plan training and continuing education; and equipment should be scheduled for calibration and maintained to ensure production quality.

## 2. Commonly used stem cell types in psoriasis

### 2.1 Adipose-derived mesenchymal stem cells

In 1973, Poznanski et al. [2] found fibroblast-like cells in fat that retained metabolic activity in vitro; Zuk (2001) [3] confirmed the existence of mesenchymal stem cells among these cells and named them adipose-derived mesenchymal stem cells (ADMSCs).

ADMSCs can be easily obtained from adipose tissue. The main method to isolate ADMSCs from adipose tissue is via collagenase digestion, which is simple to perform and produces a high yield. In addition, tissue block adherence, adsorption columns, direct centrifugation, and mechanical shaking methods are available, but efficiency is low. ADMSCs have the same regenerative properties as other mesenchymal stem cells (MSCs), which have multidirectional differentiation potential, and the potential to repair, maintain or enhance the functions of various tissues [3].

De Jesus, et al. [4] reported 1 case of psoriatic arthritis and 1 case of psoriasis vulgaris treated using autologous ADMSCs. In the former case, after ADMSC infusion, the psoriasis area and severity index (PASI) score (A measure used to assess the extent and severity of psoriasis) decreased from 21.6 to 8.9 points, but joint symptoms did not ease. After treatment with etanercept and infliximab sequentially, joint pain eased. However, psoriasis and psoriatic arthritis recurred two years later. In the latter case, other drugs were discontinued before administering ADMSC treatment, and the PASI score decreased from 24 to 8.3 after ADMSC infusion. Symptom alleviation lasted 292 days. No serious adverse events caused by ADMSC infusion were noted in these 2 patients. Amir [5] et al. reported a 6-month phase I clinical study in which ADMSCs were subcutaneously injected into the plaques of 5 psoriatic patients. After the intervention, the skin lesions of the patients all exhibited mild to significant improvement, with no major side effects, suggesting that ADMSC injection is a safe and effective method for the treatment of psoriasis.

### 2.2 Umbilical cord mesenchymal stem cells

Umbilical cord mesenchymal stem cells (UCMSCs), first reported in the early 21st century, are a type of MSC isolated from the umbilical cord and can differentiate into a variety of cells under specific conditions [6].

UCMSCs can be isolated from umbilical cord Wharton's jelly and perivascular tissues. Currently, there is no generally accepted method for isolating UCMSCs; the most used methods include the tissue explant method and enzymatic digestion. UCMSCs have strong self-renewal and proliferation abilities, a stable phenotype, and low immunogenicity [7] and are rich in content and easy to obtain [8]. Additionally, these cells are sourced from medical waste, and it is easier to obtain informed consent to authorize their reuse from an ethical standpoint; therefore, they are favored for clinical translation applications.

Cheng [9] conducted a 1/2a single-arm trial in which 17 psoriatic patients received UCMSC infusions. After treatment, The PASI scores for 47.1% of the patients improved by at least 40%, and the Physician's Global Assessment (PGA) score (a measure used by physician to assess the overall severity of patient’s condition) for 17.6% of patients suggested that the skin lesions had resolved or had almost completely resolved; no significant side effects were observed during treatment and the subsequent 6-month follow-up period. Chen et al. [10] reported that after using UCMSCs to treat 2 psoriasis vulgaris patients, skin lesions disappeared, with no recurrence for four or five years.

### 2.3 Dermal-derived mesenchymal stem cells

Dermal-derived mesenchymal stem cells (DDMSCs) are the main MSCs in the dermis and proliferate as adherent cells in vitro and have a fibroblast-like morphology [11].

DDMSCs can be isolated by tissue explant and enzymatic digestion. There are abundant tissue sources and simple isolation and culture methods for DDMSCs, and the cells have a strong proliferation ability. Campanati [12] found that the coculture of DDMSCs from healthy donors (H-MSCs) and psoriatic patients (PsO-MSCs) had positive effects on the latter, improving an inflammatory phenotype of PsO-MSCs, suggesting that DDMSCs have great potential in the clinical treatment of psoriasis.

**Table 1:** Preclinical studies of stem cell therapy for psoriasis.

Author	Sources	Location	Year
**Sah [59]**	Umbilical cord	South Korea	2016
**Rokunohe [60]**	Fat	Japan	2016
**Lee [61]**	Umbilical cord	South Korea	2017
**Kim [21]**	Tonsils	South Korea	2018
**Campanati [12]**	Dermis	Italy	2018
**Chen [34]**	Umbilical cord	China	2019
**Imai [19]**	Amniotic membrane	Japan	2019
**Kim [62]**	Embryo	South Korea	2019
**Meng [63]**	Pulp	China	2021
**Zhang [64]**	Embryo	Singapore	2021
**Chen [65]**	Umbilical cord	China	2022
**Ye [13]**	Gums	China	2022
**Zhang [55]**	Umbilical cord	China	2022
**Zhang [66]**	Umbilical cord	China	2022
**Lu [67]**	Umbilical cord	China	2022
**Ding [35]**	Umbilical cord	China	2022
**Ren [36]**	Umbilical cord	China	2023
**Wen [17]**	Pulp	China	2023
**Carrillo [68]**	Umbilical cord	Chile	2023
**Wang [33]**	Umbilical cord	China	2023

### 2.4 Gingival-derived mesenchymal stem cells

Gingival-derived mesenchymal stem cells (GMSCs) are isolated from proper gingival tissue. Similar to other MSCs, GMSCs inhibit inflammation and regulate the immune response. Under long-term culture conditions, GMSCs retain MSC characteristics, exhibit a stable morphology and retain telomerase activity [13]. GMSCs have clear advantages with regard to ethics, acquisition methods, and differentiation potential; therefore, GMSCs are considered a good source of MSCs. GMSCs can be isolated by tissue explant and enzymatic digestion. Ye et al. [13], using an imiquimod (IMQ)-induced psoriatic mouse model, found that GMSC infusion significantly alleviated psoriatic skin inflammation in mice by reducing Th1- and Th17-related cytokines.

Wang et al. [14] provided a case report on a 19-year-old male patient with severe plaque psoriasis for 5 years who experienced various local and systemic treatment failure. After receiving five injections of allogeneic GMSCs, the condition completely resolved, with no adverse reactions. There was no recurrence after 3 years of follow-up.

### 2.5 Dental pulp mesenchymal stem cells

Dental pulp mesenchymal stem cells (DPSCs), as a type of MSC in human pulp tissue, have strong proliferation, self-renewal and multilineage differentiation abilities, similar to other MSCs, as well as immune regulation and potential tissue regeneration properties [15, 16]. The methods for DPSC isolation and culture are similar to those for MSCs from other tissues.

Wen et al. [17] found that subcutaneous injection of DPSCs could reduce the symptoms of skin lesions in IMQ-induced psoriasis mouse model and suppress the expression of keratin 16, S100A8, and S100A9, which are associated with abnormal epidermal proliferation, indicating the feasibility of DPSCs for the clinical treatment of psoriasis.

### 2.6 Amniotic mesenchymal stem cells

Amniotic mesenchymal stem cells (AMSCs) are novel MSCs from the neonatal placenta and amniotic membrane, and they are perinatal stem cells with high differentiation potential. AMSCs and amniotic epithelial stem cells (AESCs) together form a thin avascular membrane called amnions. Therefore, in order to isolate AMSCs, AESCs should first be released from the amniotic membrane, followed by washing of the amniotic membrane, collagenase digestion, and cell suspension [18].

AMSCs have the same phenotypic characteristics as most MSCs, with a short spindle shape, adherent growth and tight arrangement. The cells can be passaged to P30, and changes in the morphology and proliferation ability during subsequent passages are within acceptable limits. When AMSCs are extracted in vitro, the cell acquisition rate is high, the survival rate in culture and the proliferation speed are good, which is not related to the age of the donor, and the ability of later proliferation and differentiation is relatively stable. The process of obtaining AMSCs in vitro does not harm the donor. Additionally, AMSCs have low immunogenicity in allogeneic applications, reducing the risk of rejection in allogeneic applications. From the perspective of translating experimental research to clinical application, AMSCs can be mass produced and have good stability, making them ideal target cells for stem cell research. Imai et al. [19] demonstrated that AMSCs inhibited the development of psoriatic dermatitis and attenuated the responses of keratinocytes to proinflammatory cytokines. However, clinical studies on the use of AMSCs for the treatment of psoriasis are still lacking.

### 2.7 Tonsil-derived mesenchymal stem cells

Tonsil-derived mesenchymal stem cells (TDMSCs) are MSCs from human tonsils, and similar to other MSCs, they have strong proliferation, self-renewal and multilineage differentiation abilities [20].

The isolation and culture of TDMSCs are similar to those of MSCs from other tissues. TDMSCs are usually obtained from ample sources after receiving informed consent from patients undergoing tonsillectomy and are ideal candidates for stem cell therapy. [20] . Kim et al. [21], using an IMQ-induced psoriasis mouse model, found that TDMSCs effectively inhibited the inflammation of psoriatic dermatitis. Currently, no clinical studies on the use of TDMSCs for psoriasis are available.

### 2.8 Bone marrow hematopoietic stem cells

Bone marrow hematopoietic stem cells (BMHSCs) have a high self-renewal ability and multilineage differentiation potential, can differentiate into various lineages of progenitor cells, including lymphocytes, and are the main source of all peripheral immune cells. BMHSCs can be isolated by the immunomagnetic bead method, flow cytometry and other methods from bone marrow. BMHSC transplantation is divided into allogeneic BMHSC transplantation and autologous BMHSC transplantation. Compared with autologous BMHSCs transplantation, allogeneic BMHSC transplantation is associated with a lower recurrence rate; therefore, allogeneic BMHSC transplantation has been widely used for psoriasis treatment, especially for psoriatic patients complicated with hematological diseases.

Yokota et al. reported the case of a 36-year-old male patient who had psoriasis for 25 years and suffered from aplastic anemia. After receiving allogeneic bone marrow transplantation, psoriatic symptoms completely resolved, with aplastic anemia remission [22]. Güler et al. reported the case of a 12-year-old male patient who received allogeneic bone marrow transplantation for aplastic anemia. His psoriatic lesions completely resolved, and there was no sign of psoriatic recurrence at the last follow-up on day 150 [23]. Woods et al. [24] reported the case of a 29-year-old male patient with psoriatic arthritis combined with aplastic anemia. After undergoing allogeneic bone marrow transplantation, the psoriatic condition rapidly improved, and the arthritis stabilized. The patient only had mild psoriatic symptoms on the scalp. Kojima et al. [25] reported the case of a 67-year-old male patient with psoriasis and acute myeloid leukemia. Eighteen days after undergoing allogeneic bone marrow transplantation, the psoriatic condition completely resolved, with a brief recurrence 42 days after transplantation, followed by sustained remission. Kishimoto et al. [26] reported the case of a 40-year-old male patient with acute myeloid leukemia complicated with pustular psoriasis who underwent allogeneic bone marrow transplantation and had sustained remission without relapse for 2 years. Kanamori et al. [27] reported the case of a 49-year-old male patient with psoriasis complicated with late-stage chronic myeloid leukemia. After undergoing allogeneic bone marrow transplantation, the psoriatic condition gradually resolved, and the patient was in complete remission 70 days after transplantation. His leukemia continued to be in remission. Slavin et al. [28] reported a case of psoriatic arthritis complicated with chronic myeloid leukemia; after undergoing allogeneic bone marrow transplantation, the psoriatic and arthritis symptoms were completely resolved. Chakrabarti et al. [29] reported the case of a 50-year-old male patient with generalized psoriasis combined with NHL. After undergoing allogeneic bone marrow transplantation for 21 months, psoriatic and lymphoma symptoms remained in remission.

Up to now, BMHSCs have the longest history of treating psoriasis with the highest cumulative number of cases, and UCMSCs have a single-arm study of 17 cases for psoriasis. These two kinds of cells have more data to support their effectiveness in treating psoriasis than other stem cell types. However, there are no comparative studies of the effectiveness of different stem cell types in the treatment of psoriasis (Table 2).

## 3. The scientific basis for stem cell therapy for psoriasis

An abnormal immune response activates the pathogenesis of psoriasis. The imbalanced expression of proinflammatory factors and anti-inflammatory factors, functional abnormalities of dendritic cells (DCs) and T cells, and the abnormal proliferation of keratinocytes play roles in the pathogenesis of psoriasis. DCs are critical in the initiation of psoriasis. They activate Th1 and Th17 cells to trigger downstream inflammatory responses by producing cytokines such as interleukin (IL)-23 and IL-12. IL-17 and interferon (IFN) further act on keratinocytes, forming a positive feedback loop of local inflammation [30, 31]. Compared with those in healthy people, MSCs in the lesions of psoriatic patients exhibit different characteristics: (1) higher ability to promote angiogenesis; (2) weakened antioxidant ability and immunosuppressive ability; (3) increased proinflammatory cytokine secretion and decreased anti-inflammatory cytokine secretion [32]. This may indicate that abnormal stem cells in psoriatic patients are involved in the pathogenesis of psoriasis through these three mechanisms.

**Table 2:** Case reports and clinical studies of stem cell therapy for psoriasis.

	Number of cases	Source	Autologous/allogeneic	Combination medication	Location	Year
**Jowitt [69]**	1	Bone marrow	Allogeneic	Cyclosporine	United Kingdom	1990
**Yin [70]**	2	Bone marrow	Allogeneic	/	United Kingdom	1992
**Yokota [22]**	1	Bone marrow	Allogeneic	/	Japan	1996
**Kishimoto [26]**	1	Bone marrow	Allogeneic	cyclosporine, cyclophosphamide	Japan	1997
**Cooley [71]**	3	Bone marrow	Autologous	Patient 1: cyclophosphamide, cyclosporine; Patient 2: none; Patient 3: none	Australia	1997
**Adkins [72]**	1	Bone marrow	Allogeneic	cyclosporine, methylprednisolone	United States	2000
**Slavin [28]**	1	Bone marrow	Allogeneic	Cyclosporine	Israel	2000
**Chakrabarti [29]**	1	Bone marrow	Allogeneic	Cyclosporine	United Kingdom	2001
**Kanamori [27]**	1	Bone marrow	Allogeneic	Cyclosporine, methotrexate	Japan	2002
**Kojima [25]**	1	Bone marrow	Allogeneic	Cyclosporine, methotrexate	Japan	2003
**Mohren [73]**	1	Bone marrow	Autologous	cyclophosphamide, celecoxib	Germany	2004
**Woods [24]**	1	Bone marrow	Allogeneic	cyclosporine, steroids	Canada	2006
**Masszi [74]**	2	Bone marrow	Allogeneic, autologous	Patient 1: methotrexate, cyclosporine; Patient 2: none	Hungary	2006
**Braiteh [75]**	1	Bone marrow	Autologous	None	United States	2008
**Held [76]**	1	Bone marrow	Autologous	None	United States	2012
**Mori [77]**	1	Bone marrow	Allogeneic	Cyclophosphamide	Japan	2012
**Sung [78]**	/	/	Autologous	/	United States	2015
**Chen [10]**	2	Umbilical cord	Allogeneic, autologous	None	China	2016
**De Jesus [4]**	2	Fat	Autologous	Patient 1: etanercept, infliximab; patient 2: oral hydroxychloroquine, topical clobetasol propionate, petrolatum jelly, betamethasone valerate	Philippines	2016
**Azevedo [79]**	1	Bone marrow	Autologous	/	Portugal	2017
**Comella [80]**	1	Fat	Autologous	/	United States	2018
**Chen [81]**	1	Bone marrow	Autologous	/	China	2018
**Seetharaman [54]**	1	Fat	Allogeneic	/	India	2019
**Ciurea [82]**	6	Bone marrow	Allogeneic	Methotrexate, tacrolimus, rabbit anti-thymocyte globulin	United States	2019
**Wang [14]**	1	Gums	Allogeneic	None	United States	2020
**Ugur [83]**	4	Bone marrow	Autologous	/	Turkey	2021
**Dilara [23]**	1	Bone marrow	Allogeneic	Cyclosporine, methotrexate, cyclophosphamide	Turkey	2021
**Yao [45]**	7	Fat	Autologous	Urea ointment	China	2021
**Chen [9]**	17	Umbilical cord	Allogeneic	None	China	2022
**Amir [5]**	5	Fat	Allogeneic	None	Iran	2023

A series of studies have investigated the mechanism of stem cell therapy in psoriasis. Most studies have focused on UCMSCs. In vivo studies have shown that UCMSCs reduce inflammatory cytokines, including IFN-γ, tumor necrosis factor (TNF)-α and IL-17A, in psoriatic patients [9, 33]. Additionally, Th1/Th17 cells, which play key roles in the pathogenesis of psoriasis, also decrease after the application of UCMSCs [9, 33]. While Treg cells and CD4^+^ memory T cells show increasing trends, studies have shown that the baseline level of Treg cells is a sign of the response or nonresponse of patients to UCMSC treatment and that baseline Treg cell levels in responders are lower [9]. In the IMQ-induced psoriasis mouse model, UCMSCs exhibit corresponding therapeutic effects [34]. In addition, UCMSCs play roles by inhibiting the local infiltration of neutrophils [34, 35] and intervening in keratinocyte proliferation through MMP13 [36]. ADMSCs have been found to reduce inflammatory cells and inflammatory factors that play key roles in the pathogenesis of psoriasis [4, 5, 37]. BMMSCs and ADMSCs can relieve psoriatic skin inflammation in mice by upregulating TGF-β and inhibiting keratinocyte proliferation [37]. Studies have shown that TDMSCs relieve psoriatic skin inflammation in mice mainly by attenuating the Th17 response in a PD-L1-dependent manner [21]. DDMSCs inhibit the proliferation of CD3^+^ T cells, promote their apoptosis, and reduce the Th17/Treg ratio, possibly by upregulating TGF-β [38].

In summary, different types of stem cell therapy have enormous potential for the treatment of psoriasis through the inhibition of immune cell proliferation and differentiation, proinflammatory factor expression, and keratinocyte proliferation.

## 4. Institutional requirements for the preparation of stem cells and related products

Stem cell product preparation at institutions involves biomedicine, ethics, laws and regulations, among other fields. The core requirements are as follows.

### 4.1 Ethics and compliance

Researchers should conduct an ethical review for stem cell research to ensure compliance with the "Measures for the Ethics Review of Biomedical Research Involving Humans", and the source and acquisition process of stem cells should be ethical.

### 4.2 Quality management

The preparation of stem cells requires the establishment of a quality control and assurance system, including cell characterization, sterile technique, and environmental monitoring.

4.2.1

The preparation should follow GMPs and relevant regulations, and a complete quality management system should be established.

4.2.2

Institutions must perform risk assessments and reasonably design work areas to ensure that the functional areas are independent and meet requirements.

4.2.3

Control the microorganism, particulate and pyrogen contamination risk.

4.2.4

Management and quality control personnel must have relevant professional knowledge and experience and cannot serve concurrently. All relevant personnel must receive professional training.

4.2.5

Personnel should report possible contamination in a timely manner and take measures to avoid contamination.

4.2.6

The traceability of equipment and instruments must be ensured, and management will be performed in accordance with requirements detailed in instruction manuals.

4.2.7

If an electronic information system is used, institutions should establish and verify relevant management procedures.

### 4.3 Intellectual property rights

When considering innovation and invention in the stem cell field, attention should be given to intellectual property rights, such as patent protection.

## 5. Stem cell collection, preparation, storage, and quality control

### 5.1 Collection of stem cells

Standard operating and management procedures for stem cell collection, isolation and stem cell culture must be formulated and strictly implemented on the basis of meeting GMP requirements. During the collection, isolation and culture of stem cells, autologous stem cells that have not been subjected to complex in vitro manipulations should be identified and tested for viability, proliferation ability, exogenous pathogenic microorganisms, and basic stem cell characteristics. For allogeneic stem cells that have undergone complex in vitro manipulations, in addition to the abovementioned assessments, comprehensive detection of endogenous pathogenic microorganisms, detailed detection of stem cell characteristics, and cell purity analysis should also be performed.

### 5.2 Preparation and processing of stem cells

The stem cell preparation process includes the collection, isolation, purification, expansion and passaging, directed differentiation toward functional cells, selection criteria and use of media, excipients and packaging materials, cryopreservation, recovery, aliquoting, labeling of cells, and residue removal. An SOP for the stem cell preparation process and an SOP for each process must be formulated, periodically reviewed and revised.

After stem cells enter the human body, they face the pathological microenvironment caused by disease, which can lead to oxidative stress in and apoptosis of the transplanted cells and damage the inherent therapeutic characteristics of the stem cells. Therefore, over the past few decades, researchers have worked to fine-tune the properties of stem cells for harsh pathological environments to make them more suitable for specific diseases. Based on the plasticity and memory ability of stem cells, cues in the wound microenvironment, such as environmental factors (hypoxia) [39, 40] and chemical factors (inflammatory factors and cytokines) [41, 42], will be the main consideration when handling stem cells in vitro. There is a growing body of evidence that indicates that properly priming stem cells with disease-related stimuli can improve their biological function and thus yield a better therapeutic effect. For example, the proliferation, migration, and survival rates of TNF-α-preactivated MSCs significantly increase under hydrogen peroxide-induced oxidative stress.

### 5.3 Storage of stem cells

High-quality and stable storage of stem cell resources is a key factor in the application of stem cells. Low-temperature cryopreservation technology provides a vital guarantee for the transportation, long-term storage, and activity and function preservation of stem cells. Different types of stem cells have different low-temperature biological characteristics and thus different abilities to cope with cryo-damage. Therefore, the application of unique optimal cryopreservation methods to preserve different stem cells can ensure the safety and effectiveness of thawed stem cells.

### 5.4 Quality control for stem cells

The inspection of stem cell preparations can be divided into quality inspections and release inspections. Quality inspections are comprehensive assessments conducted to ensure the safety, effectiveness and quality controllability of stem cells after specific in vitro treatments. Release inspections are relatively fast and simplified cell assessments that should be performed for each batch of stem cells prepared before clinical application, after the quality inspection is completed. Quality inspections include cell identification; assessments of viability, growth activity, purity and homogeneity; sterility and mycoplasma detection; endogenous pathogenic factor detection; endotoxin detection; assessments of abnormal immunological responses and tumorigenicity; biological efficacy tests; and the detection of residual amounts of media and other added components. Project applicants must develop release inspection items and standards based on the characteristics of each type of stem cell prepared using the inspection content and standards specified in the abovementioned quality inspection items. A professional cell inspection institution/laboratory will perform quality inspections of stem cell preparations and issue inspection reports.

In addition to meeting the above quality inspection requirements for stem cell preparations, at each stage of preclinical and clinical research, a comprehensive safety, efficacy and stability study of stem cell preparations should be conducted using different in vitro methods, including assessments of growth activity and status, tumorigenicity and tumor promotion, and biological effects, as should a study of the stability of stem cell preparations during storage (cryopreservation in liquid nitrogen and temporary storage of cells before implantation) and after transportation. The test items should include cell viability, density, purity, and sterility. Based on the results of the stability assessments, the components and formulation of the preservative solution, the storage and transportation conditions, and the expiration date of the preparations, as well as the transportation containers and means compatible with the expiration date and qualified facilities and conditions for cell cryopreservation, should be determined.

## 6. Methods, doses, and routes of stem cell therapy for psoriasis

**Figure 1.**
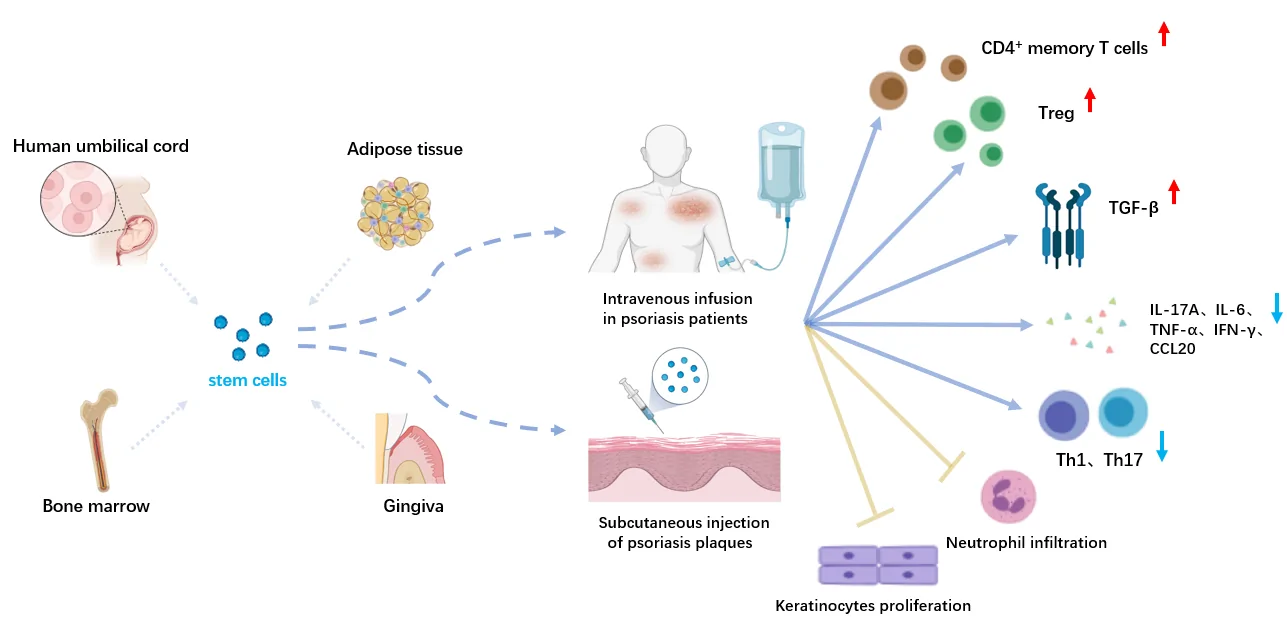
Stem cells in the treatment of psoriasis.

At present, the existing domestic and international stem cell therapies for the treatment of psoriasis mainly include the intravenous infusion of BMHSCs, intravenous infusion or subcutaneous injection of ADMSCs, intravenous infusion of UCMSCs, and intravenous infusion of GMSCs (Fig. 1).

### 6.2 Dosage and route of stem cell therapy for psoriasis

6.2.1

Intravenous infusion of BMHSCs: At present, there are no clear recommendations for a fixed mode of administration and dose for the use of BMHSCs for the treatment of psoriasis. Previous studies have reported on intravenous infusion in patients with hematological diseases complicated with psoriasis; however, the dose and the number of infusions were different. The methods for allogeneic/autologous BMHSC transplantation therapy were as follows: (1) selective infusion of CD34^+^ cells (0.5~5) × 10^6^ cells/kg to (1.5~2.5) × 10^7^ cells/kg, or the total number of cells was (2~4) × 10^8^ cells/kg after a single infusion; and (2) antihistamine used before infusion.

6.2.2

Methods for the treatment of ADMSCs: (1) The recommended intravenous infusion dose is 0.5~3×10^6^ cells/kg once every 4 weeks for a total of 2 to 3 infusions. (2) For the subcutaneous injection of ADMSCs, a single dose should be injected into the subcutaneous tissue of each plaque, with a total cell number of 1 × 10^6^ or 3 × 10^6^ cells/cm^2^.

6.2.3

Intravenous infusion of UCMSCs: The recommended dose is (1.5-2) × 10^6^ cells/kg once every 2 weeks for a total of 4 infusions; or (2.5-3) ×10^6^ cells/kg once every 4 weeks, for a total of 2 infusions; or 1 × 10^6^ cells/kg, 1 to 3 infusions within 3 weeks, and another 2 infusions after 12 weeks, depending on the situation.

6.2.4

Intravenous infusion of GMSCs: Clinical cases of GMSCs are rare. The recommended dose in previous international cases was 3 × 10^6^ cells/kg, twice every week. After 5 weeks, the second course of infusion can be performed, depending on the situation.

General rules for the intravenous infusion of stem cells: (1) Before infusion, each bag of cells should be gently inverted several times to make the suspension even. (2) A blood transfusion filter is recommended for infusion, as is a thick vein in the patient’s arm for infusion. (3) Intravenous infusion should begin slowly (20-30 drops/min) for 10 min. If there is no notable reaction after 10-15 min, the drip rate can be adjusted to 40-50 drops/min until the infusion is complete; if the patient has cardiovascular disease, the drip rate should be controlled in accordance with the doctor’s advice. (4) After all the cells are infused, the pipeline should be rinsed with 30-50 mL of normal saline.

Published studies show that there are differences in the types of stem cells used and the drug delivery methods. Because of differences in isolation sources and drug delivery methods, the efficacy and safety of stem cells in the treatment of psoriasis also vary. In conclusion, the application of stem cells has not yet been standardized, and the administration methods, dosage, and duration of treatment in the treatment of psoriasis still need to be studied in-depth.

## 7. Management of stem cell infusion for the treatment of psoriasis: conditioning, monitoring, and treatment of adverse reactions

### 7.1 Pretreatment

On the day of treatment, the stem cells should be thawed and ready to be administered. Each batch of stem cells should undergo release inspection before transplantation. Stem cells should be suspended in 5 mL of normal saline before injection [14]. For local injections, stem cells were resuspended in 0.9% saline at a density of 3 × 10^6^ cells/mL. For intravenous infusion, stem cells were resuspended in 0.9% saline at a density of 1 × 10^6^ cells/mL [43]. Currently, there are no clinical studies on the preconditioning of stem cells for the treatment of psoriasis. In the existing clinical trials of stem cell therapy for psoriasis, to prevent the occurrence of adverse reactions, antihistamine, such as diphenhydramine, is usually injected intramuscularly before infusion. For the treatment of psoriasis using stem cells, the conditioning regimens need to be further studied, and the various methods and combinations used need further optimization.

### 7.2 Monitoring

During the infusion, changes in patient respiration, pulse, blood pressure, and blood oxygen saturation should be closely monitored by electrocardiogram (ECG), and first aid measures should be prepared. A few patients may experience transient chest tightness, fever and other discomfort that will generally resolve on their own. If the symptoms persist or are severe, treatment should be stopped, vital signs should be taken, ECG and other examinations should be completed, and necessary first aid measures such as oxygen inhalation should be implemented.

### 7.3 Handling of adverse reactions

Based on studies published in Stem Cell Reports on the treatment of other diseases and clinical study data and case reports for the treatment of psoriasis [4, 9, 14, 43-45], no serious adverse events have been observed in clinical applications. The reported safety events were mainly low fever, chest tightness, dizziness, mild abdominal pain, abnormal ECG, and pharyngitis. [9, 45] and usually resolved within 24 h. Individual patients may have a high fever, for which physical cooling and symptomatic drug treatment can be given. Additionally, the possibility of an infusion reaction and anaphylactic shock cannot be excluded, and active symptomatic treatment should be performed once such events occur.

## 8. Clinical evaluation of stem cell therapy for psoriasis (efficacy and safety)

In clinical studies, the PASI score is often used to evaluate the severity of skin lesions before and after treatment. The PASI score is a comprehensive evaluation of the area, erythema, scale and thickness degree of lesions. The main assessment sites are the head, trunk, upper limbs, and lower limbs. PASI 75 represents a 75% improvement in the PASI score after treatment compared with that at baseline, with PASI 90, PASI 100, and so on, indicating improvements of 90%, 100%, etc. [46].

Based on comprehensive clinical studies and case reports published on stem cell therapy in the field of psoriasis, therapy can be divided into three-time windows: treatment period, remission period, and relapse. During treatment, the rate of improvement in the PASI score was the main evaluation indicator in 2 clinical studies, and one case report evaluated the changes in the absolute value of the PASI score during treatment [9, 45]. While evaluating improvements in skin lesions, the change trend for PGA, Dermatology Life Quality Index (DLQI), and Itch Numeric Rating Scale (INRS) scores after treatment can be simultaneously evaluated from the perspectives of the doctor and the patient [4]. In the remission stage, the skin lesions of psoriatic patients gradually resolved. A number of studies have reported that the skin lesions of psoriatic patients completely cleared after stem cell therapy, that is, a PASI 100 response rate [10, 14, 26, 27, 43, 47]. In addition, three studies confirmed that after stem cell treatment, psoriatic lesions basically cleared; that is, a PASI 90 response rate or PGA 0/1 score were achieved [9, 48, 49]. Although previous studies have confirmed the effectiveness of stem cell therapy, a number of studies have noted the issue of recurrence after stem cell therapy, with observation time windows ranging from 2 to 5 years. For the definition of recurrence, the currently accepted standard is improvement in PASI score ≤50% of the baseline PASI score, and the time to relapse is the time from the cessation of treatment to a reduction in PASI improvement by 50% [4, 47, 50]. Therefore, when evaluating the efficacy of stem cell treatment for psoriasis, the PASI improvement rate or PASI absolute value score is recommended as the main evaluation index during the treatment period, and the PASI 75/90/100 is recommended as the main evaluation index in the remission stage. Attention should be given to recurrence and the median time to recurrence in patients under long-term observation. Because psoriasis is a systemic disease, tools such as the Psoriatic Arthritis Disease Activity Score (PASDAS) and global patient assessment are recommended for evaluating the overall condition of the patients [51].

Among the reported clinical data for stem cell treatment of psoriasis and other diseases, there have been no serious adverse events. Nevertheless, the safety of stem cells during and after treatment still cannot be ignored. First, in the preparation process, the selection of donors and tissues and the purity and tumorigenicity of stem cells need to be considered [52]. Second, cell clumps should be avoided during stem cell treatment. During treatment, attention should be paid to the total number of cells in a single infusion, and the infusion rate should be controlled to avoid the most dangerous complication: pulmonary embolism. In short-term safety evaluations after treatment, two clinical studies found that fever was the most common adverse reaction [53]. Some case reports included patients with other tumor-related diseases [10, 26] and the patients were immunocompromised and at risk of infection. Another case report found that after a patient with palmoplantar pustulosis was in complete remission after receiving stem cell therapy, he developed other autoimmune diseases, such as autoimmune thyroiditis, 5 months later [26]. Therefore, during the development of clinical trials, vital signs should be closely monitored, routine tests (routine blood tests, routine urine tests, and routine stool tests) should be performed, and organ function, immune function, pathogens, inflammation (erythrocyte sedimentation rate, C-reactive protein, etc.), infection, laboratory indicators such as ECG and adverse events such as allergic reactions, organ damage, and tumors should be assessed.

## 9. Applied research on stem cell derivatives

Stem cell derivatives are bioactive components released or secreted into the extracellular matrix by stem cells and include stem cell conditioned medium (CM) and extracellular vesicles (EVs) [54]. Stem cell CM contains proteins, microRNA, growth factors, antioxidants and other active substances secreted by stem cells. Stem cell EVs include exosomes, microbubbles, apoptotic bodies, etc. The existing studies of EVs related to the treatment of psoriasis mainly use exosomes.

Zhang [55] et al. found that the subcutaneous injection of UCMSC-derived EVs (50 μg) effectively reduced the levels of proinflammatory cytokines and chemokines (including IL-17, IL-23, TNFα and CCL20) in an IMQ-induced psoriasis mouse model and inhibited the activation of DCs through the inhibition of the JAK-STAT pathway in mice. The subcutaneous injection of IFNγ-preconditioned EVs (150 μg) from UCMSCs also relieved psoriatic dermatitis in mice, suggesting that EVs secreted by IFN-γ-stimulated UCMSCs are promising cell-free therapeutics. Growth factors, anti-inflammatory factors, chemokines, cytokines and other paracrine factors are contained in stem cell CM [56]. CM from stem cells may directly act on epidermal cells and promote the formation of extracellular matrix, thus helping skin regeneration and improving psoriasis [54].

Stem cell derivatives have also been successfully used in clinical research. Seetharaman [54] et al. topically applied ADMSC-derived conditioned media (ADMSC-CM) to psoriatic lesions once a day for a month to treat a 38-year-old psoriasis vulgaris patient. The PASI score decreased from 28 points to 0 points, and quality of life improved, with no adverse reactions. In addition, Wang [57] proposed that UCMSC-derived conditioned medium (UC-CM) has low cytotoxicity, good anti-inflammatory ability, and the ability to regulate metabolism and that UC-CM has therapeutic potential for psoriatic patients.

Although stem cell derivatives have not been widely used in clinical research, compared with stem cells, their derivatives have the following characteristics: (1) easier to store and transport; (2) fewer ethical issues, as they are noncell biological therapies; (3) non-self-replicating, thus avoiding the risk of carcinogenesis; and (4) low immunogenicity and low allergenicity and thus safer than stem cells. These characteristics suggest that there is great potential for stem cell derivatives in the treatment of psoriasis.

## 10. Conclusion and prospects

As cells in the human body with various differentiation potentials and immune regulation, stem cells have been explored as options for the treatment of psoriasis for many years, and the underlying mechanisms have been studied. Stem cells are expected to be used as a new method for the treatment of psoriasis. Different types of stem cells have been found to correct the unbalanced immune response and improve clinical symptoms of psoriasis by regulating the infiltration of immune cells and the levels of inflammatory factors in psoriatic skin lesions. Stem cell derivatives, such as growth factors and exosomes secreted by stem cells, are cell-free therapy methods and have various application advantages, such as convenient acquisition, stable properties, easy storage, and fewer ethical concerns; therefore, there is potential for these derivatives to be promoted in clinical practice. In addition, the methods of combining stem cells with drugs, Chinese medicines and small molecule compounds have also been investigated. For example, studies have shown that quercetin, a Chinese medicine ingredient, can promote the anti-inflammatory effect of UCMSCs in the treatment of rheumatoid arthritis [58]. Therefore, it is conceivable to optimize the combination of various methods to maximize the therapeutic effect of stem cell therapy for psoriasis. It is worth noting that in the commercialization process of stem cell technology, the complex challenges related to patent rights and intellectual property require special attention. Despite the innovative therapeutic approaches brought about by stem cell research and development, finding a balance between protecting patents, ensuring the fairness of technology transfer, and adhering to ethical standards represents a critical issue that must be addressed by research institutions and commercial entities. In conclusion, as an emerging method in the treatment of psoriasis, stem cells and their derivatives have enormous potential for future applications.
